# Correlation analysis of resected breast tissue and implant volume after mastectomy and its association with breast density

**DOI:** 10.1007/s00404-021-06128-1

**Published:** 2021-06-29

**Authors:** Wolfram Malter, Bo Jan Bachmann, Barbara Krug, Martin Hellmich, Max Zinser, Peter Mallmann, Christian Eichler, Julian Puppe

**Affiliations:** 1 Department of Gynecology and Obstetrics, Medical Faculty, University of Cologne, Cologne, Germany; 2 Department for Diagnostic and Interventional Radiology¸ Medical Faculty, University of Cologne, Cologne, Germany; 3 Institute of Medical Statistics and Computational Biology, Medical Faculty, University of Cologne, Cologne, Germany; 4 Department for Plastic and Reconstructive Surgery, Medical Faculty, University of Cologne, Cologne, Germany

**Keywords:** Implant volume, Breast density, Resected breast tissue, Implant-based breast reconstruction

## Abstract

**Background:**

The current methods for calculating the ideal implant volume for breast reconstruction are based on pre- or intraoperative volume measurements of the existing breast volume and do not take into account the individual breast density of the woman. This study aims is to identify objective parameters that can help to improve the optimal implant selection.

**Materials and methods:**

This retrospective analysis includes 198 breast cancer patients who underwent mastectomy. Breast densities (ACR) measured in mammography and MRI were compared with the removed breast tissue weight and volume of the implants used. In addition, the resected weight was compared directly with the implant volume to calculate a mathematical function.

**Results:**

There was no significant correlation between the ACR values and the resected weights [correlation coefficient: mammography:− 0.117 (*p* = 0.176), MRI − 0.033 (*p* = 0.756)]. A negative correlation between the implant volumes and both imaging methods could be demonstrated [correlation coefficient: mammography − 0.268; *p* = 0.002; MRI was − 0.200 (*p* = 0.055)]. A highly significant correlation between the resected weights and the implant volumes (correlation coefficient 0.744; *p* < 0.001) was observed. This correlation corresponds to a power function (*y* = 34.71 *x*^0.39^), in which any resected weight can be used for the variable *x* to calculate the implant volume.

**Conclusion:**

We were able to show that there is a significant correlation between the resected breast tissue and the implant volume. With our novel potency function, the appropriate implant volume can be calculated for any resected weight making it easier for the surgeon to choose a fitting implant in a simple and more objective manner.

## Background

Breast cancer is still the most common malignant tumor in women worldwide [1]. About every eighth woman will develop breast cancer in the course of her life [2]. Surgical intervention, systemic therapy and radiation therapy and are the three pillars of breast cancer treatment. Today, in almost 80% of patients the tumor can be removed by breast-conserving surgery. However, a mastectomy, in which the entire mammary gland is removed, may still be unavoidable for certain indications [3, 4].

This intervention can have significant consequences for the women concerned. In addition to physical impairments, psychosocial consequences, such as the feeling of a lack of femininity, can also be stressful. Breast reconstruction is therefore of essential importance in today’s oncoplastic breast surgery [5, 6]. Breast reconstruction can be done with the help of the body’s own tissue or an artificial implant. When it comes to implant reconstruction, the selection of the right implant is crucial with regard to the functional and cosmetic result. In addition to standardized distance measurements, impression procedures or radiological imaging procedures, tissue-based planning can be used for implant selection [7, 8]. Here, the observed dimensions and measurements guides the appropriate choice of the breast implant [8]. Since objective selection criteria for optimal implant selection have not yet been sufficiently evaluated, many surgeons rely on their knowledge and subjective experience [9].

The aim of our retrospective study is to identify objective parameters that can positively influence decision-making in favor of the optimal implant selection. To answer this question, we analyzed the correlation between the weight of the removed mammary tissue and the used implant volume. Moreover, the weight of the resected tissue and the volume of the implant were compared with the breast gland density measured by radiological imaging. Based on this data, we would like to provide breast surgeons with a new kind of calculation tool that could facilitate the selection of the implant and improve the functional and cosmetic result.

## Materials and methods

### Data acquisition

The data collection was based on the oncological documentation program ODSeasy^®^ (Asthenis^®^ GmbH, Aschheim, Germany) of the clinic and polyclinic for gynecology and obstetrics at the University of Cologne. Patient that received both a mastectomy and subsequently a breast implant between Jan 1, 2006, and Jan 31, 2018, were included in this retrospective study. Clinical parameters including TNM classification, hormone receptor status, tumor grading, indication and type of ablation were collected from patient’s files. The weight of the resected breast tissue was taken from the pathology report. The implant volume and breast density in mammography and MRI were found in the surgical and radiology report, accordingly. The resected weight contains the sum of the main operation and all documented surgical specimens of reoperations for incomplete tumor excision.

### Inclusion and exclusion criteria

All patients had to have received a mastectomy followed by an implant reconstruction between Jan 1, 2006, and Jan 31, 2018, were included in the study. Breast-conserving therapy that had previously taken place did not lead to exclusion. Pre- or postoperative measures such as chemotherapy, radiation or endocrine therapies, as well as missing MRI or mammography findings did not constitute an exclusion criterion. The exclusion criteria were defined as information on the weight of the resected breast specimen, no information on the implants used, missing written pathological reports and patients who had undergone radical mastectomy without breast reconstruction or expander insertion.

### Statistical procedure

The data collected were evaluated using the SPSS version 25 statistical software (IBM Corp., Armonk, NY, USA). Here, the breast densities (ACR) measured in mammography and MRI were compared on the basis of their four-stage division, both with the removed resected weight and with the volume of the implants used. In addition, the weight of the resected breast tissue was compared directly with the implant volume.

The Spearman rank correlation coefficient was calculated. This correlation coefficient gives a measure of the (monotonous) relationship between two variables. Its absolute values can vary between − 1 and + 1. Positive values correspond with positive correlations and vice versa values close to 1 indicating a high correlation and values close to zero pointing to a nonexistent correlation. All analyses are essentially explorative with *p* values ≤ 0.05 (*) indicating moderate evidence, *p* values ≤ 0.01 (**) intermediate evidence and *p* values ≤ 0.001 (***) strong evidence against the null hypothesis (e.g., zero correlation). The number of patients (Valid N), the mean value (Mean), the median, the standard deviation, the minimum and maximum as well as the 25th and 75th percentile were given in each comparison that we carried out.

The comparison between resected weight and implant volume was also shown in a potential function. The course of the function shows both disproportionate and disproportionate increases between the two variables, as well as the extent to which individual values deviate from the curve.

## Results

**Fig. 1:**
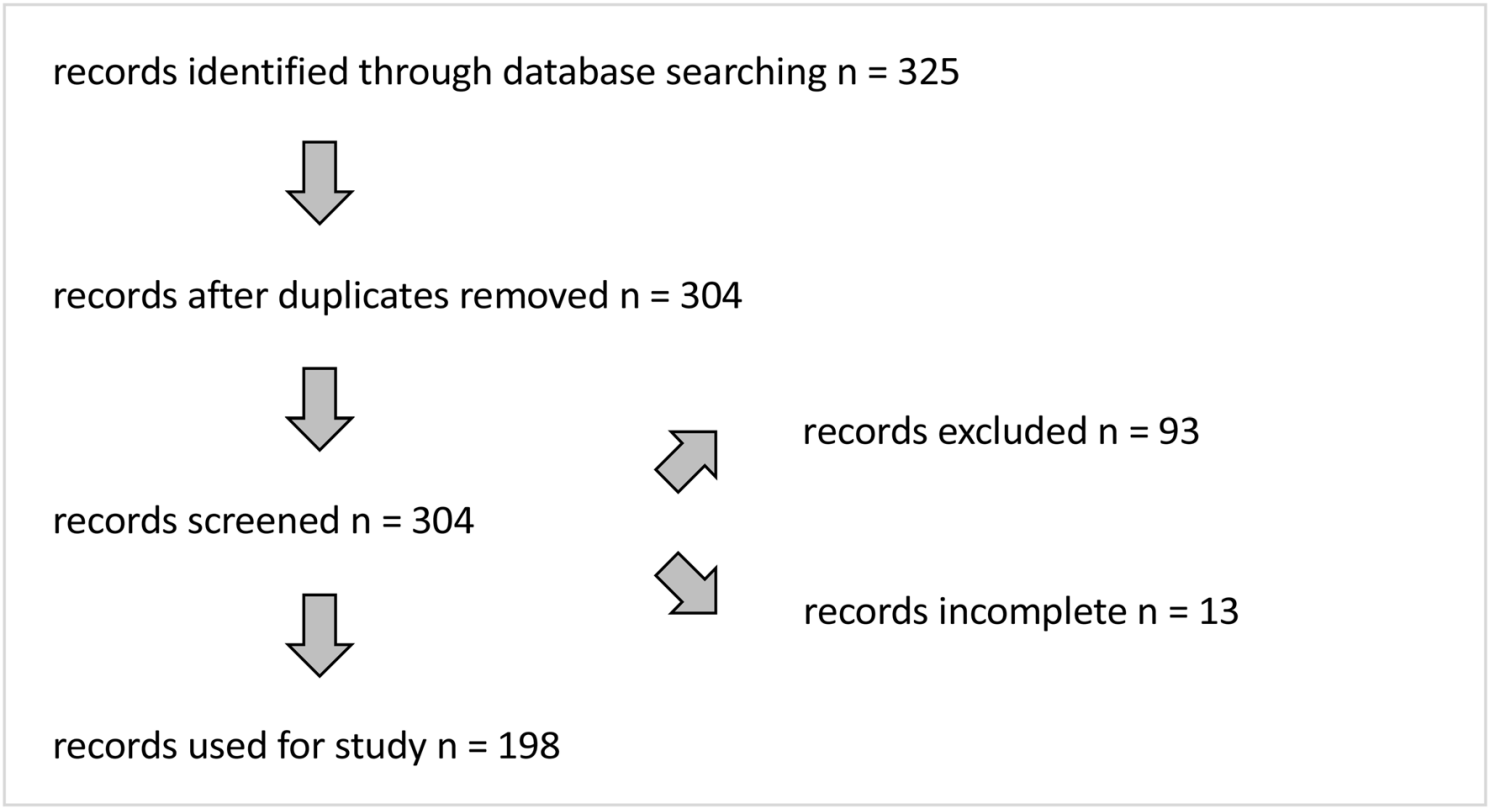
Schematic representation of the inclusion and exclusion of patient data

A total of 325 patient cases were identified based on the inclusion and exclusion criteria. Mastectomies on both sides made it possible for individual patients to be listed twice. Therefore, in 21 cases, the data were combined for one patient case. Cases that involved radical mastectomy without breast reconstruction or expander insertion were excluded from the data collection. This applied to a total of 93 patients. In addition, a further 13 cases were not taken into account in which essential data such as the pathology reports were missing. A total of 198 patient cases with the associated data formed the study cohort (Fig. 1). The patient characteristics of the study cohort are summarized in Table 1.

**Table 1:** Patient characteristics of the study cohort

	Valid *n*	%
Age (y)		
Overall average	49	
Menopause status (*n*)		
Premenopausal	109	55.1
Perimenopausal	13	6.6
Postmenopausal	75	37.9
Not specified	1	0.5
Tumor stadium (*n*)		
Tis	56	28.3
T0	28	14.1
T1	73	36.9
T2	33	16.7
T3	8	4.0
Nodal status (*n*)		
N0	154	77.8
N1	30	15.2
N2	6	3.0
Nx	8	4.0
Metastatic status (*n*)		
M0	131	66.2
M1	2	1.0
Mx	65	32.8
Grading (*n*)		
G1	15	7.6
G2	105	53
G3	64	32.3
Not specified	14	7.1
BCT before ablation (*n*)		
No (primary)	165	83.3
Yes (secondary)	33	16.7
Indication ablation (*n*)		
DCIS	73	36.9
IDC	99	50.0
ILC/ITC	20	10.1
Preventive ablation	3	1.5
Others	3	1.5
Type of ablatio (*n*)		
SSM	54	27.3
NSM	144	72.7
Implant location (*n*)		
Epipectoral	117	59.1
Subpectoral	81	40.9
Implant side (*n*)		
Left	99	50.0
Right	99	50.0
Contralateral side (*n*)		
No	130	65.7
Yes	68	34.3

### Comparison of breast density (ACR mammography/MRI) with the weight of the resected breast tissue

**Fig. 2:**
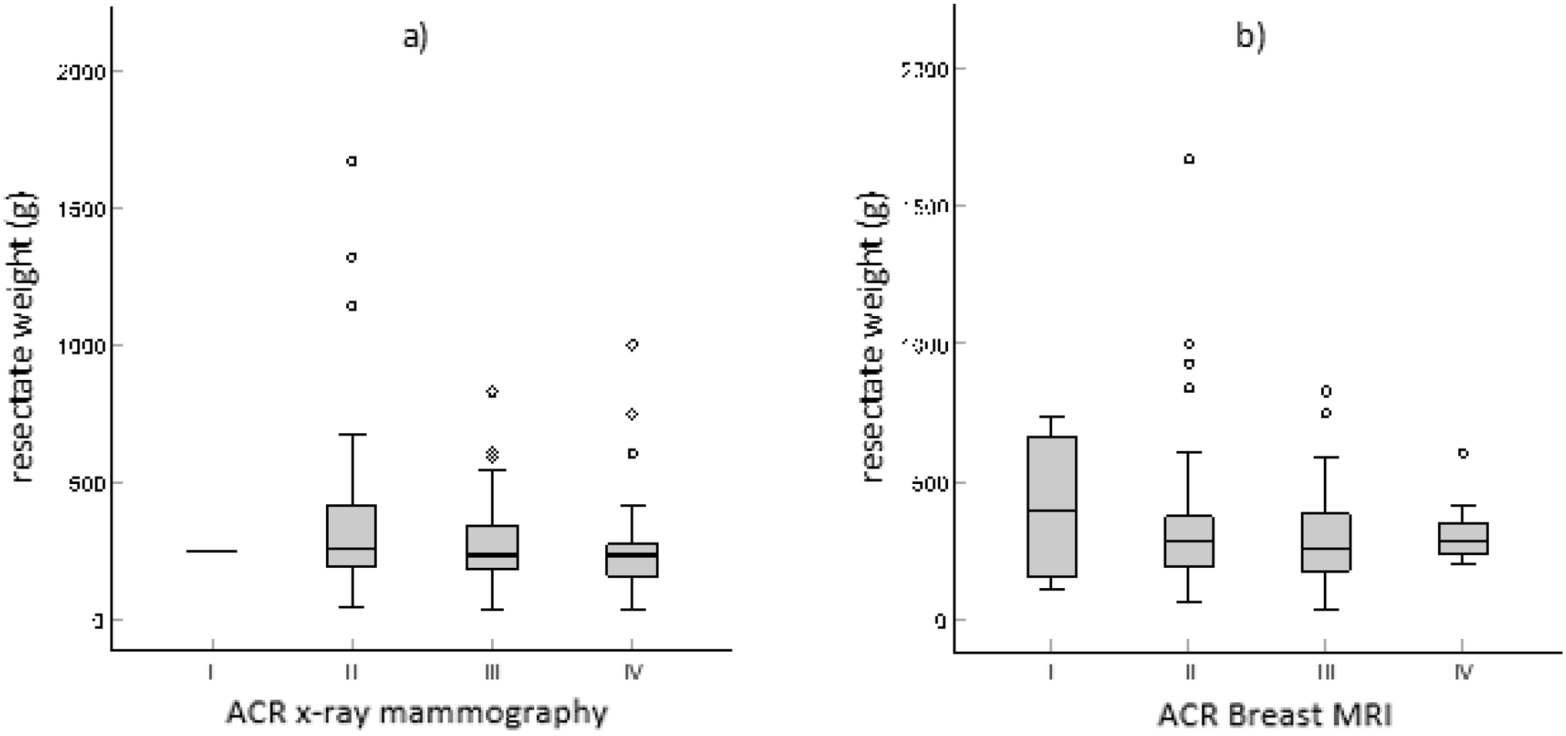
Resected weights and breast density (ACR values I-IV) with **a** mammography and **b** MRI

First of all, we examined whether there was a relationship between the weight of the resected tissue and the density of the mammary gland measured in radiological imaging. For this purpose, the weight of the resected specimen was compared to the individual breast density (ACR). Table 2 and Fig. 2 show the distribution of the resected weights on the four ACR values in mammography and MRI.

**Table 2:** Distribution of the resected weights within the four ACR values

		Resected weight of breast tissue (g)														
		Valid *n*	Mean		Standard deviation		Minimum		Percentile 25		Median		Percentile 75		Maximum	
ACR mammography	I	1	251,00				251,00		251,00		251,00		251,00		251,00	
	II	38	374,37		337,59		41,00		188,00		253,50		417,00		1670,00	
	III	69	273,16		144,93		39,00		179,00		240,00		341,00		830,00	
	IV	27	271,11		213,14		33,00		121,00		239,00		275,00		1000,00	
ACR MRI	I	4	410,75		302,00		113,00		156,50		395,00		665,00		740,00	
	II	49	353,57		272,06		67,00		195,00		281,00		373,00		1670,00	
	III	30	300,47		189,96		39,00		179,00		256,50		382,00		830,00	
	IV	10	320,80		119,48		202,00		239,00		285,50		351,00		606,00	

The table shows the number of patients (Valid N), the mean value (Mean), the median, the standard deviation, the minimum and maximum as well as the 25th and 75th percentile

As we expected, there was no significant correlation between the ACR values and the resected weights. The correlation coefficient in mammography was − 0.117 (*p* = 0.176), in MRI it was − 0.033 (*p* = 0.756) (Table 4). Therefore, based on the preoperatively determined breast gland density (ACR), no conclusions can be drawn about the expected resected weight.

### Comparison of breast density (ACR mammography/MRI) with the implant volume used

**Fig. 3:**
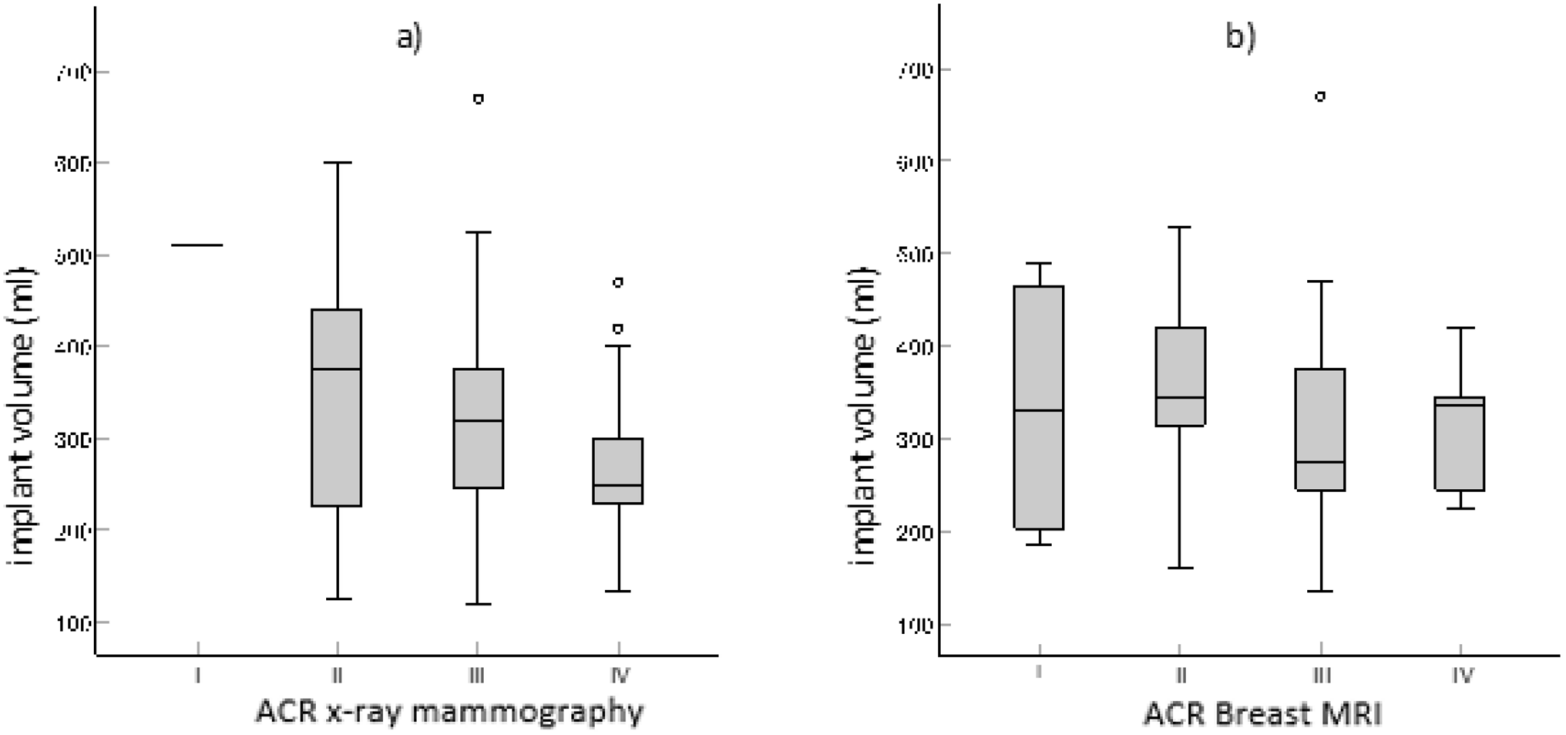
Implant volumes and breast density (ACR values I-IV) with **a** mammography and **b** MRI

To determine whether the implant selection could be made easier on the basis of the preoperatively determined breast gland density, the ACR value in mammography or MRI was compared with the implant volume used (Fig. 3) (Table 3). Mammography showed a significant negative correlation (correlation coefficient of − 0.268; *p* = 0.002). The correlation coefficient for MRI was − 0.200 and thus reflects a trend (*p* = 0.055) (Table 4). Therefore, an increasing breast density is associated with a smaller implant volume independent of the weight of the resected breast tissue. Overall, mammography was superior to MRI.

**Table 3:** Distribution of implant volumes within the four ACR values

			Implant volume (ml)												
		Valid *n*		Mean		Standard deviation		Minimum		Percentile 25		Median		Percentile 75	Maximum
ACR mammo- graphy	I	1		510,00				510,00		510,00		510,00		510,00	510,00
	II	38		343,68		119,77		125,00		225,00		375,00		440,00	600,00
	III	69		314,49		103,61		120,00		245,00		320,00		375,00	670,00
	IV	27		267,41		79,41		135,00		225,00		250,00		300,00	470,00
ACR MRI	I	4		333,75		153,59		185,00		202,50		330,00		465,00	490,00
	II	49		353,27		94,45		160,00		315,00		345,00		420,00	530,00
	III	30		310,83		123,18		135,00		245,00		275,00		375,00	670,00
	IV	10		315,50		62,51		225,00		245,00		335,00		345,00	420,00

The table shows the number of patients (Valid N), the mean value (Mean), the median, the standard deviation, the minimum and maximum as well as the 25th and 75th percentile

**Table 4:** Correlation coefficient of implant volumes, resected weights, ACR mammography and ACR-MRI, in each case

		Implant volume (ml)	Resected weight (g)	ACR mammography	ACR MRI
Implant volume (ml)	Correlation coefficient	1.000	0.744^a^	− 0.268^a^	− 0.200
	Sig. (2-tailed)		< 0.001	0.002	0.055
	*n*	198	198	135	93
Resected weight (g)	Correlation coefficient	0.744^a^	1000	− 0.117	− 0.033
	Sig. (2-tailed)	< 0.001		0.176	0.756
	*n*	198	198	135	93
ACR mammography	correlation coefficient	− 0.268^a^	− 0.117	1000	0.449^a^
	Sig. (2-tailed)	0.002	0.176		0.000
	*n*	135	135	135	73
ACR MRI	Correlation coefficient	− 0.200	− 0.033	0.449^a^	1.000
	Sig. (2-tailed)	0.055	0.756	0.000	
	*n*	93	93	73	93

^a^Correlation is significant the 0.01 level (2-tailed)

*Sig.* significance

### Comparison of the weight of the resected breast tissue with the inserted implant volume

Finally, we analyzed the extent to which the resected weight of the breast tissue correlates with the selected implant volume regardless of the radiological density (ACR). Here a highly significant correlation between the resected breast tissue and the implant volumes could be demonstrated (correlation coefficient 0.744; *p* < 0.001) (Table 4). To make it easier for the surgeon to choose the implant based on this observation, we were looking for a mathematical application. It was calculated that this correlation corresponds approximately to a potential function. It can thus be the potential function.

*y* = 34.71 *x*^0.39^

**Fig. 4:**
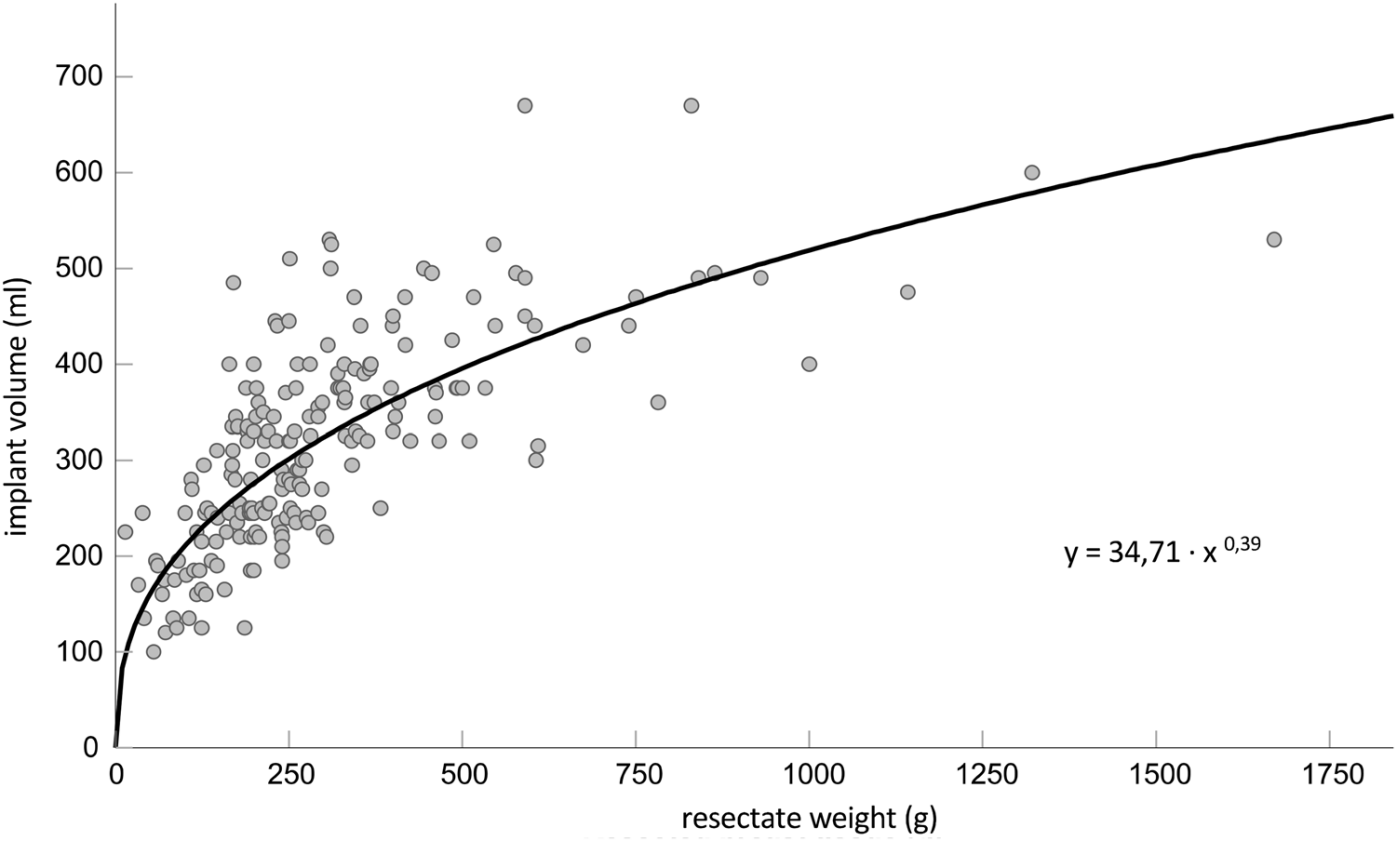
Correlation between resected weight of breast tissue and implant volume per case. The curve shows the potential function *y* = 34.71 *x*^0.39^

**Fig. 5:**
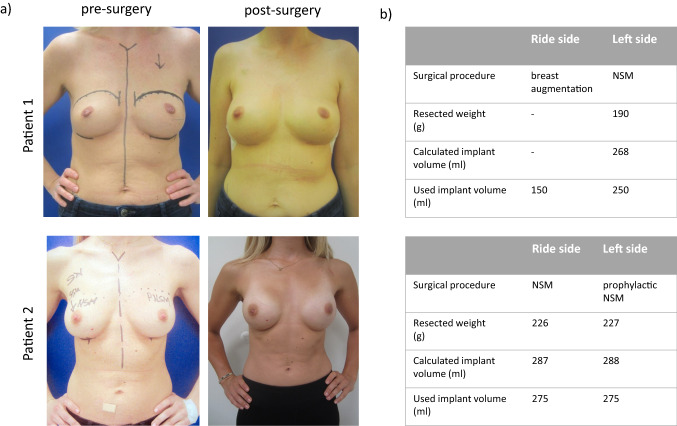
**a** Representative cases of breast cancer patients showing the pre- and postoperative outcome after mastectomy (*n* = 2). **b** Table shows the correlation between the calculated implant size using the potential function and used breast implant volume. (*NSM* prophylactic nipple-sparing mastectomy)

In which any resected breast tissue weight can be inserted for the variable *x* and thus an implant volume can be calculated (Fig. 4). As proof of principle, we tested this formula in two patient cases. Here we could demonstrate that the calculated implant size correlates with the inserted implant volume (Fig. 5).

## Discussion

Oncoplastic breast reconstruction is now generally accepted as an important part of the operative treatment of breast cancer patients. The goal here is to restore an aesthetic breast shape and symmetry. There is no recognized standard method for the selection of the appropriate implant [10, 11]. The numerous techniques for pre- and intraoperative estimation of the implant size are often imprecise and prone to errors [9, 12]. The preoperative estimation procedures include anthropometric volume estimation and volumetric analysis using ultrasound, mammography, computer tomography, magnetic resonance imaging and three-dimensional scanners. In addition, the thickness of the residual skin covering after mastectomy cannot be correctly estimated using this method [9, 11].

One of the intraoperative estimation techniques is the “implant size method”, in which implant prostheses of different sizes are used one after another on a trial basis and thus gradually approach the ideal. This method is easy to implement, but costly because many implant sizes have to be stored in the operation room. In the “gauze swab implant estimation method”, the required volume is estimated using gauze swabs soaked in sodium chloride and also inserted into the implant pocket on a trial basis. Deviations in size and properties between the different manufacturers of the gauze can falsify this inexpensive method [9, 12].

In addition, the final decision in favor of an implant is subjectively influenced in many cases by the experience, personal preferences and surgical skills of the surgeon [9].

All of the methods mentioned for determining the breast volume are based on the same principle of determining the volume as accurately as possible by means of measurement technology directly on the individual, pre- or intraoperatively.

Our approach is based on the assumption that the ideal implant size can be derived from the ratio between the resected weight and the implant volume. In practice, it is obvious that the selected implants became larger as the amount of resected tissue increased. Our study confirms this observation. We were able to show that there is a significant correlation between the resected breast tissue and the implant volume. From this, we were able to derive a potential function. With this function, the appropriate implant volume can be calculated for any resected tissue directly in the OR, making it easier for the surgeon to select an optimal fitting implant.

We also examined whether breast density has an impact on the weight of the resected tissue and the volume of the implant. The idea is that higher densities in the tissue might result in higher weights. Thus, a breast with a higher percentage of parenchyma with the same volume should be heavier than one with a high percentage of fat. With breast reconstructions, however, it is assumed that the weight of the breast tissue corresponds to the volume of the breast tissue. Applied to reconstructive breast surgery, it would therefore be expected that a lower breast density is associated with a relatively smaller resected tissue weight, but must be compensated for with more implant volume. We were able to confirm this relationship on the basis of our data. It was shown here that with increasing ACR, i.e., higher breast density, a smaller implant was chosen. This observation is supported by a recent South Korean study. Here the actual volume was measured using the Archimedes principle based on the water displacement of resected breast tissue in saline solution. The study was able to show that the displaced volume decreases with increasing breast density [13]. This correlation appears to be independent of the weight of the resected tissue since we could not find any significant connection between the ACR value and the weight of the resected tissue. Presumably, this could be explained by the observation that dense breasts contain less fat tissue and therefore might be smaller.

Using the newly generated formula, a more precise assessment of the implant size can then be made intraoperatively. Thus, the surgeon has a new tool for an objective pre- and intraoperative implant selection. The advantage compared to already established methods is the simple and quick implementation. There are hardly any costs because fewer implant sizes have to be kept available. In addition, subjective and therefore error-prone factors such as the surgeon's aesthetic preference based on a mathematical calculation of the implant selection have less influence.

This study has some limitations and further studies with higher numbers of cases are necessary to evaluate the clinical benefit in the routine. In particular patients with hypo- and macromastia were underrepresented in this study. For this subgroup, the formula might be inaccurate due to the fact that some patients with small breasts request a larger implant or a breast reduction surgery in case of macromastia. The formula might not be useful for the reconstruction of ptotic breasts since many patients might need a bigger implant than the volume to achieve a satisfactory cosmetic outcome. Furthermore, the informative value for patients with a low breast density is limited, since the group ACR I was underrepresented in this evaluation. Due to the retrospective nature of the study, no data on the postoperative aesthetic outcome could be collected. In this regard, further studies should check whether the result obtained with this formula for surgery achieves the same patient satisfaction compared to established methods and, if necessary, could even improve it for inexperienced surgeons. A practical application of our approach would be the future integration of the formula into software that could be used at any time on-site using a smartphone or computer.

## Conclusion

Overall, we were able to generate a formula by inserting the weight of the resected breast tissue for a better assessment of the implant size. This formula can be used intraoperatively. Presumably, this could make it easier for the surgeon to choose the optimal fitting implant.
